# The Physiology of the Fœtus, Liver, and Spleen

**Published:** 1831

**Authors:** 


**The Physiology of the Fœtus, Liver, and Spleen.** By *George Calvert Holland*, M.D. Lecturer on Physiology, and Joint Lecturer on the Practice of Physic in the Sheffield Medical Institution, &c. &c. Octavo, pp. 229. London, 1831.

Dr. Holland's quarry has not been low, in aiming at the physiology of the fœtus, liver, and spleen. It would be difficult to select subjects on which more has been said than on these, and on which more evanescent theories have been advanced. The profession look with a certain degree of jealousy on treatises concerning the office of the spleen, and were we booksellers we should hesitate, we should "pause and ponder, and ponder and pause," before we ventured on any *splenetic* speculation. In truth, Dr. Holland's title is not a taking one. A recipe for a new liniment, a specific for a sore leg, would be a much more popular affair than the most recondite discoveries in physiology. The public appear to us to think that physiologists do often play them false, and the multitudes of physiological pretenders of late years has undoubtedly injured the whole fraternity; for when much bad metal is known to be current, honest folks frequently refuse the good. The fact is, that plain persons have been not a little puzzled. One man makes an experiment and deduces from it a certain conclusion; another repeats it and draws precisely the opposite inference. Conflicting statements arise at every turn of the road, till plain practical people get fairly bothered and will listen no more. In former times the error of physiologists appeared to be an excessive disposition to theorize; at the present it seems to consist in an indiscriminate rage for experiments. The following remarks on the subject by Dr. Holland are pertinent.

> "In investigating the operations of nature, the inquiring mind is perplexed by studying them through the medium of these ever varying systems, which involve the most important subjects of medical and physiological inquiry in almost impenetrable obscurity. There are scarcely any truths in medicine, like the axioms in geometry, or the first principles in philosophy, so universally allowed, and fully established, that the student of this science can rely on their correctness; it is, therefore, absolutely requisite, amidst so many discrepant theories, clashing opinions, and opposite conclusions, drawn from the same experiments, to put every thing to the test of the most elaborate and tedious examination. Another cause, which has retarded the progress of medical knowledge, is *an almost exclusive attention to experiments*.
>
> However valuable the important discoveries which have been made by a recurrence to experiments, much error has arisen from the false inferences that have been frequently drawn from them,—inferences which, though really the mistakes of erroneous observation, have been regarded as incontrovertibly correct.
>
> It is very allowable to doubt the truth of propositions supported by argument alone, but it seems an act of bold temerity to call in question facts, or obvious deductions from them, said to be proved by direct experiment; and hence, whilst the former are fearlessly controverted, the latter are generally received with too easy an assent." *Introd.* xx.

The first chapter is on the physiology of the liver and spleen. Before propounding his own theory respecting these organs, Dr. Holland endeavours to demolish the theories of his predecessors. With regard to the liver he disproves, or rather attempts to disprove, the idea that the bile is secreted by the vena portæ. His reasons are these:—that all other secretions are performed by arteries; that in Mr. Abernethy's case there was no vena portæ, and yet bile was formed; and that in all the molluscæ the liver is very large, and is supplied from the aorta only. We conceive that it would not be difficult to answer these objections.

"From what has been previously explained, it is evident that the hepatic artery is regarded as the source of bile; and believing the production of this to be only one function of the liver, it is my intention to account for the great quantity of venous blood transmitted to it and the spleen. Both organs are well adapted to receive a great share of sanguineous fluid, whether we consider the texture as composed of blood-vessels or of cells.

The function of the spleen, as well as that of the liver, independently of the secretion of bile, is considered a *diverticulum* of the system. *If the veins which form the vena porta had passed directly to the vena cava inferior, a thousand accidents, arising either from mental or corporeal disturbance, would have continually placed the life of an individual in imminent danger. Every passion, whether of an exciting or depressing character, and every general and local disease, if severe, whether chronic or acute, would have been liable to have deranged the lungs and heart.* I have already endeavoured to shew, that passions, of a depressing nature, bring the blood in greater quantity than usual from the inferior and superior extremities, and also from the surface of the body to the chest; and I have also stated, that the abdominal viscera participate in the engorgement.* Since the body is continually liable to such changes, baneful effects would follow, unless nature had provided organs, whose situation, function, and organization, enable them to *diminish the burden imposed upon those, whose constant and almost regular action is indispensable for the maintenance of life.* This object is beautifully answered by the liver and spleen. The organs within the chest must be regarded as possessing vital functions. If the lungs were surcharged with blood, or in a condition approaching to it, the properties and distribution of this fluid would be immediately disordered, and with this primary derangement every part of the body would quickly sympathize.

*The liver and the spleen, from being placed close to the thorax, are calculated to relieve the congested lungs and heart, or rather to protect them from sudden and violent commotions;* and are also favourably situated to protect in the same manner the stomach, whose action is scarcely less vital." 20.

Such is Dr. Holland's explanation of the liver and spleen; and after the note of preparation sounded, we must own that we expected something more. The *diverticulum* theory is somewhat older than the Doctor; and the only remark we think it necessary to offer is, that he, in our opinion, attributes to it an undue importance. Our author bears hard on the assumptions of others; yet, if our readers will refer to the present work, they will find, we believe, that his arguments are chiefly assumptions. The second portion of the volume is on the physiology of the fœtus. The following appears to be his theory of the materno-fœtal vascular connexion.

"The venous blood transmitted by the fœtus to the placenta, is absorbed by veins and conveyed to the lungs of the mother; the arterial blood derived from the uterus, is absorbed by the umbilical vein, and flows to the heart of the fœtus. We are not capable of forming an accurate idea of the number or nature of those powers which cause, in either case, the return of blood to the heart. It is quite clear, however, that it flows to the placenta from two opposite points, and

* "See Experimental Enquiry, Chap. xv."

that it is absorbed and carried to the mother and fœtus in a similar way, although not precisely the same, in both instances. The views of Barry go far towards explaining the circulation of venous blood in viviparous animals after birth, but do not account for its circulation in the fœtus.

It was stated by Vieussens that the red globules of blood were not absorbed by the umbilical vein. The same absurd doctrine was also taught by Monro, Primus. 'The fœtuses of viviparous animals,' observes the latter, 'have their red blood from the same source that chickens in eggs have theirs, *the action of the heart and the vessels in their body and secundines*.'*

If oxygen, and that organic construction universally belonging to the respiratory organs of animal and vegetable life be wanting, the process of oxygenation cannot possibly proceed. The simple action of muscles, or vessels, on the particles of blood, cannot create an arterial fluid.

I have already alluded to the impossibility of the placenta performing the office of lungs. If a connexion between the placenta and uterus either of a *direct* or *indirect* kind be denied, it is impossible to account for the origin of fœtal blood. The fœtus possesses not the organs necessary to produce it, and, therefore, it must necessarily pass from the uterus to the placenta." 115.

Thus, he considers it impossible for the fœtus to make blood, because it has no organs of digestion to form chyle, the pabulum, nay the very essence of this fluid, no organs of respiration to oxygenate the chyle when formed. The separation of the placenta, without hæmorrhage after the birth of the child, he believes to be effected by the engorgement of the placenta, consequent on the destruction of its connexion with the umbilical vein—the strong contractions of the uterus exerted upon the placenta—the effect of those contractions in diminishing the capacity of the uterine arteries, and thus directly lessening the connexion of the placenta with the uterus, and also preventing the occurrence of hæmorrhage. Such are the views of Dr. Holland, and for the reasoning by which they are supported we refer to the work itself. As we have not leisure for a critical examination of the hypothesis, we will not offer an ex cathedrâ opinion on its merits.

The fœtus has been said or proved to possess a lower temperature than the maternal system; it seldom exceeds 92° or 95° F. The temperature of the fœtus lying dead in the uterus, is said, on the contrary, to be higher. Magendie has attempted to account for this by supposing that the fœtus in utero has some means of lowering its temperature; a very unlikely supposition. We think, *if the fact be a true one*, that the imperfect oxygenation of the blood in the fœtus would account for it. Dr. Holland is of opinion that the distance of the fœtal system from the centre of the circulation, the passage of the blood from the uterine arteries of the mother into the umbilical veins of the fœtus, should be regarded as the causes of the phenomenon. He accounts for the living fœtus being colder than the dead one, by the *cooling* processes of circulation, assimilation, and secretion, going on in the former. We were not previously aware that all these were cooling processes, and in truth we look upon the whole of the statements on the subject with suspicion.

The next subject which engages the attention of Dr. Holland is the origin of the liquor amnii and meconium. For one who is ever inveighing against the disposition of the world to receive theories rather than to sift their pro-

* "Edinburgh Medical Essays, Vol. II. p. 301."

per pretensions, we must say that the absence of every thing like positive facts from his chain of reasoning, is surprising. We again refer our readers to the work itself in proof of our assertion. Dr. Holland is far from timid in reasoning on assumptions.

"If we allow that the amnion secretes, the activity of its function will be admitted to be proportionate to its vital energies; but this activity cannot rationally be supposed to equal that of the well-known powers of the skin, which are engaged, in every period of life, in removing from the surface of the body, perspirable matter. The physiologist may very safely infer, that the amnios is secreted by the skin, from the fact, *that such secretion is its natural and unceasing function, and is, moreover, a necessary consequence of its organization, and the distribution of blood to it.* That it is secreted by the amnion, is very improbable. If the fœtus had been placed in a medium of a temperature much lower than its own, *transudation* from the surface of its body would have been almost wholly prevented; but, as the fluid, in which it is immersed, is of the same temperature as itself, such an effect is rather facilitated than impeded.

'Deux causes,' observes Edwards, 'influent principalement sur ces deux fonctions: (absorption and transudation) la quantité de liquide contenue dans le corps, et le température de l'eau dans lequel il séjourne. Plus il y a de plénitude, moins il y aura d' absorption; plus la température de l'eau est basse, moins il y aura d' exsudation. Dans la limite extrême de 30° cent. le contraire a lieu; les pertes l'emportent de beaucoup sur les gains, parceque la transsudation prédomine sur l'absorption.'*

The liquor amnii augments with the development of the fœtus. If this fluid is exuded from the skin, its gradual increase is in perfect accordance with those principles which are supposed to explain its origin. *The vital actions of the fœtus, and a more extended surface for secretion, augment in proportion to the progress of gestation.*" 135.

The meconium is referred to the secretions of the abdominal viscera, totally independent of any digestive process. In a chapter on the nutrition and peculiarities of the fœtus, our author comes to the following conclusion.

"I am not, I believe, too sanguine in my anticipations, when I state, that an acquaintance with these truths, will completely subvert the present doctrines of nervous affections; the prevalent ideas respecting the cause of animal temperature; the common notions of the susceptibility of the body to morbid action; and the views generally taken of the origin of acute and chronic diseases, as well as many other corporeal derangements." 148.

It has been a disputed point with physiologists, whether the blood in the umbilical vein differs from that in the umbilical arteries. Dr. Holland thinks he has set the point at rest in the affirmative by an experiment.

"It has already been stated, in the preceding pages, that the umbilical vein carries arterial blood from the placenta to the fœtus. This opinion is supported by Drs. Bostock and Jeffrey. Physiologists in general are either opposed to this conclusion, or consider it doubtful.† It is really astonishing, that a subject so easy to investigate, and to decide by experiment, has been allowed to remain so long undetermined. From the kindness of my friend Mr. Carr, surgeon,

* "De l'Influence des Agens Physiques, p. 352."

† "Bichat, treating of this subject, observes, 'Les faits précédens suffisent au reste pour établir, comme un fait incontestable, l'uniformité du sang des deux systèmes chez le fœtus."

Sheffield, I have been enabled to prove, by an experiment of the simplest kind, that the umbilical vein circulates arterial blood. The following was the method employed by Dr. Jeffrey :—He took part of the cord, and dissected away the gelatinous substance of it, until he had laid bare the vessels, when, on puncturing them, he observed that there was a difference of colour between the blood in the vein and the arteries. Many physiologists are said to have failed in this experiment. From the tedious preparation which it required I did not succeed to my full satisfaction. But on taking part of the cord, as soon as the child was born, around which I had previously tied a ligature, about two or three inches from the free extremity, and cutting this with a sharp scalpel, in order to make an even surface, I very clearly discerned, on pressing the cord from below upwards, blood of a very different colour, flowing from the umbilical vein and arteries. If the experiment does not fully succeed in the first instance, tie a ligature still farther removed from the end, and, having made a fresh surface, press gently from below upwards. Sometimes a large drop of florid blood is observed to stand directly over the umbilical vein, and another dark coloured over the arteries, without their being in the least mingled with each other, and, in this case, the difference between the two is so striking that no one can fail to observe it. This experiment being so simple, and requiring no previous preparation, any individual of the most ordinary powers, or unscientific character, may easily perform it." 154.

He thinks that the frequency of the heart's action in the fœtus, will tend to render the blood less black for the following reasons.

"The whole mass of the blood of an adult circulates throughout the system once every three minutes, because the heart contracts about 75 times per minute; if it contracted oftener with equal or superior energy, the same quantity of blood would complete its revolution in considerably less time. The heart of the fœtus, instead of contracting 75 times per minute, contracts, at least 120 times. In considering this subject, we must not forget, *that there is the same relation between the heart of the fœtus and the quantity of blood belonging to its body, as there is between that of the adult and the quantity of blood circulating in its system.*

It is therefore obvious, if the fœtal heart contracts 120 times per minute, that the complete circulation of the blood in its system will be effected in almost half the time of that of the adult, and consequently, although venous blood is always mingling with the arterial fluid of the umbilical vein, the blood of the fœtus cannot be particularly deteriorated from this circumstance, because the whole mass is frequently renewed by the very numerous contractions of the heart. If this contracted 75 times instead of 120, the relation between it and the mass of the circulating fluid remaining the same, the arterial qualities of the blood would be diminished almost one-half." 156.

In the eighth chapter Dr. Holland endeavours to prove that the brain, the spinal cord, the stomach, the liver, the pancreas, and the intestines are not essential to fœtal life. In the ninth, he considers the uses of the suprà renal capsules, the thymus, and the thyroid glands in the fœtus, and decides that they are *diverticula* to the organs in their proximity. In the tenth chapter Dr. Holland points out the differences in the mode of nourishment between the oviparous, and ovo-viviparous, and viviparous animals. The last chapter is devoted to the influence of the imagination of the mother on the development and constitution of the fœtus. Into all these points our readers will hold us excused for not entering. We have given a sufficient number of extracts to enable the public to form an opinion respecting the manner and merits of Dr. Holland's essay. For our own parts, we unhesitatingly assert that it is distinguished by a great superfluity of theory, and an equal deficiency of tangible facts. The author has been free in censuring the world for their disposition to believe on slight grounds, or no grounds at all, and if he has laid himself open to a similar charge, he must not be annoyed or astonished, if the world, through our mouths, retort. Yet, in justice to Dr. Holland, we must say that he evinces, on many occasions, considerable ingenuity, a fair acquaintance with the rules of argument, and we think his criticisms frequently alike just and shrewd. Dr. Holland may complain that we pronounce a dogmatic opinion on the work; that we condemn without shewing fair grounds for our condemnation. To this we reply, that we have done Dr. Holland more justice than ourselves, for our aim and object have been to shew his opinions and say very little respecting our own. The extracts which we have given will in some measure enable our readers to determine, whether our remarks are founded in justice or not.

